# SAMHD1 Enhances Chikungunya and Zika Virus Replication in Human Skin Fibroblasts

**DOI:** 10.3390/ijms20071695

**Published:** 2019-04-05

**Authors:** Sineewanlaya Wichit, Rodolphe Hamel, Andreas Zanzoni, Fodé Diop, Alexandra Cribier, Loïc Talignani, Abibatou Diack, Pauline Ferraris, Florian Liegeois, Serge Urbach, Peeraya Ekchariyawat, Andres Merits, Hans Yssel, Monsef Benkirane, Dorothée Missé

**Affiliations:** 1 Department of Clinical Microbiology and Applied Technology, Faculty of Medical Technology, Mahidol University, Nakhon Pathom 73170, Thailand; 2 Laboratoire MIVEGEC, UMR 224 IRD/CNRS/UM1, 34394 Montpellier, France; rodolphe.hamel@ird.fr (R.H.); fodediop@hotmail.com (F.D.); loic.talignani@ird.fr (L.T.); diack89@yahoo.fr (A.D.); pauline.ferraris@ird.fr (P.F.); florian.liegeois@ird.fr (F.L.); 3 Aix-Marseille Université, Inserm, TAGC UMR S1090, 13288 Marseille, France; andreas.zanzoni@inserm.fr; 4 Institut de Génétique Humaine, CNRS-Université de Montpellier, UMR 9002, 34090 Montpellier, France; alexandra.cribier@igh.cnrs.fr (A.C.); monsef.benkirane@igh.cnrs.fr (M.B.); 5 Institut de Génétique Fonctionnelle, Functional Proteomics Platform, 34090 Montpellier, France; serge.urbach@fpp.cnrs.fr; 6 Department of Microbiology, Faculty of Public Health, Mahidol University, Bangkok 10400, Thailand; peeraya.ekc@mahidol.ac.th; 7 Institute of Technology, University of Tartu, 50090 Tartu, Estonia; andres.merits@ut.ee; 8 Centre d’Immunologie et des Maladies Infectieuses, Inserm, U1135, Sorbonne Universités, UPMC, APHP Hôpital Pitié-Salpêtrière, 75013 Paris, France; hans.yssel@inserm.fr

**Keywords:** SAMHD1, arbovirus, Chikungunya, Zika, IFIT, SILAC

## Abstract

Chikungunya virus (CHIKV) and Zika virus (ZIKV) are emerging arboviruses that pose a worldwide threat to human health. Currently, neither vaccine nor antiviral treatment to control their infections is available. As the skin is a major viral entry site for arboviruses in the human host, we determined the global proteomic profile of CHIKV and ZIKV infections in human skin fibroblasts using Stable Isotope Labelling by Amino acids in Cell culture (SILAC)-based mass-spectrometry analysis. We show that the expression of the interferon-stimulated proteins MX1, IFIT1, IFIT3 and ISG15, as well as expression of defense response proteins DDX58, STAT1, OAS3, EIF2AK2 and SAMHD1 was significantly up-regulated in these cells upon infection with either virus. Exogenous expression of IFITs proteins markedly inhibited CHIKV and ZIKV replication which, accordingly, was restored following the abrogation of IFIT1 or IFIT3. Overexpression of SAMHD1 in cutaneous cells, or pretreatment of cells with the virus-like particles containing SAMHD1 restriction factor Vpx, resulted in a strong increase or inhibition, respectively, of both CHIKV and ZIKV replication. Moreover, silencing of SAMHD1 by specific SAMHD1-siRNA resulted in a marked decrease of viral RNA levels. Together, these results suggest that IFITs are involved in the restriction of replication of CHIKV and ZIKV and provide, as yet unreported, evidence for a proviral role of SAMHD1 in arbovirus infection of human skin cells.

## 1. Introduction

Arbovirus infection causes an increasing global burden for public health. Although the distribution of many arboviruses is geographically restricted, they can unexpectedly spread outside their area of endemicity and the past five decades have seen an unprecedented emergence of epidemic arboviral diseases. In this respect, there has been a major geographical expansion of Chikungunya virus (CHIKV) and Zika virus (ZIKV) in Central and South America [1].

CHIKV is a member of the *Togaviradae* family, belonging to the genus alphavirus, that is present in tropical and subtropical regions [2]. Lately, it has spread to the Western hemisphere and is now endemic in the Caribbean islands and South and Central America including Mexico [1]. Up to ninety percent of infected individuals develop Chikungunya fever, characterized by high fever, myalgia, joint pains, rash, and intense asthenia [3]. Frequently long-term complications such as encephalopathy, encephalitis, myocarditis, hepatitis, and circulatory failure do occur [4,5].

ZIKV belongs to the genus flavivirus of the *Flaviviridae* family [6]. The largest ZIKV pandemic occurred in Brazil in 2015 and subsequently spread throughout the Americas infecting nearly two million people [7]. The clinical presentation of ZIKV infection range from asymptomatic to a flu-like illness. More recently, however, an increase in the incidence of Guillain-Barré syndrome, among infected adults, and neurological birth defects, including microcephaly, in newborn babies have been reported [8] and corroborated by the observation that ZIKV is a teratogenic agent able to cross the placental barrier to cause severe neurological damage in developing fetuses [9,10].

CHIKV and ZIKV are predominantly transmitted by *Aedes* (*Ae.*) *aegypti* and *Ae. albopictus* mosquitoes [11], although evidence of sexual transmission and blood transfusion-related transmission of ZIKV have been reported [12,13]. Human infections with arboviruses occur during blood feeding by infected mosquitoes. During blood meals, the mosquito׳s mouthpiece is introduced into the skin, resulting in extravascular delivery of infectious viral particles in both the epidermis and dermis where resident and migratory cells encounter the pathogen [14,15].

To gain more insight into the complex interactions between CHIKV, ZIKV and the skin, we determined the proteome profile of human skin fibroblasts during viral infection, using Stable Isotope Labeling by Amino acids in Cell culture (SILAC)-based Liquid Chromatograph-tandem Mass Spectrometer (LC-MS/MS) analysis. Previous studies have focused on the proteome profile on CHIKV infection of neuronal cells, myocytes and hepatocytes and identified several differentially expressed proteins involved in stress response, cytoskeleton/cell structure, transport/trafficking and signaling [16,17,18,19]. However, neither of the cells represents a major entry site for CHIKV and ZIKV, unlike the skin [20,21].

In the present study, we analyzed the proteome changes during CHIKV and ZIKV infection of human fibroblasts with the aim of identifying novel molecular processes involved in replication of these arboviruses. We report the identification of 16 differentially expressed proteins following infection of human skin fibroblasts with either CHIKV- or ZIKV-infected cells, including interferon (IFN)-stimulated proteins and defense response proteins. Moreover, we show that the Sterile Alpha Motif (SAM) domain and histidine/aspartic acid (HD) domain containing protein 1 (SAMHD1) enhanced both CHIKV and ZIKV replication, while IFN-induced proteins with tetratricopeptide repeats (IFITs) may be inhibitory to virus replication.

## 2. Results

### 2.1. Identification of Differentially Expressed Proteins in Infected HFF1 Cells

To analyze changes in proteome response to viral infection, HFF1 cells were grown in either light or heavy labeled medium and infected with CHIKV or ZIKV. The cells were harvested for nanoflow LC-MS analysis after 48 h post infection (hpi). Light and heavy mock-infected cells were used as negative control. Proteomic analysis resulted in the identification of 2132 and 2696 differently expressed proteins in CHIKV- and ZIKV-infected cells, respectively (Tables S1 and S2). The expression of sixteen proteins was found to be significantly up-regulated in either CHIKV- or ZIKV-infected cells, nine of which were common in both CHIKV- and ZIKV- infected cells (Table 1).

**Figure 1:**
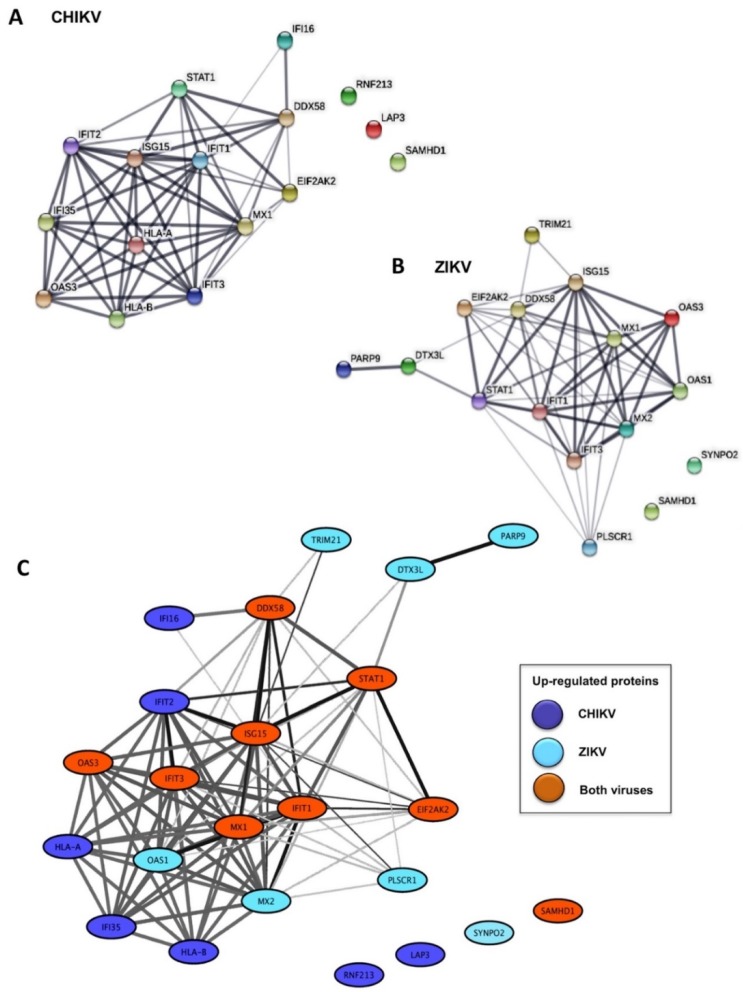
Network representation of proteins significantly up-regulated in CHIKV and ZIKV-infected HFF1 cells: (**A**) functional interaction network among significantly up-regulated proteins in CHIKV-infected cells; (**B**) functional interaction network among significantly up-regulated proteins in ZIKV-infected cells; and (**C**) merged functional interaction network among all significantly up-regulated proteins in both CHIKV- and ZIKV-infected cells. Proteins are represented as nodes and functional relationships by edges. The thickness of edges is proportional to the confidence level of the functional relationship.

Following a functional enrichment analysis, seventeen and eleven annotations were found to be significantly enriched among up-regulated proteins in CHIKV-infected cells and ZIKV-infected cells, respectively (Table 2 and Table S3). The enriched functional annotations for both CHIKV and ZIKV virus-infected cells included proteins involved in the Type-I Interferon (IFN-I) signaling pathway, anti-viral defense responses to virus, negative regulation of the viral infection process and viral genome replication (Table 2 and Table S3). Most of these proteins are encoded by interferon-stimulated genes (ISGs) whereas several represent IFN-induced proteins with tetratricopeptide repeats (IFITs), including IFIT1 (ISG56), and IFIT3 (ISG60). The functional relatedness of these arbovirus-induced proteins is corroborated by their high interconnectivity in the STRING human functional interaction network (Figure 1A,B). Interestingly, nine identical closely related proteins (MX1, IFIT1, IFIT3, ISG15, DDX58, STAT1, OAS3, EIF2AK2 and SAMHD1) were up-regulated in both infection models and, with the exception of SAMHD1, all were interconnected in the functional network (Figure 1C).

### 2.2. Expression of IFIT Proteins Affects CHIKV and ZIKV Replication in HFF1 Cells

**Figure 2:**
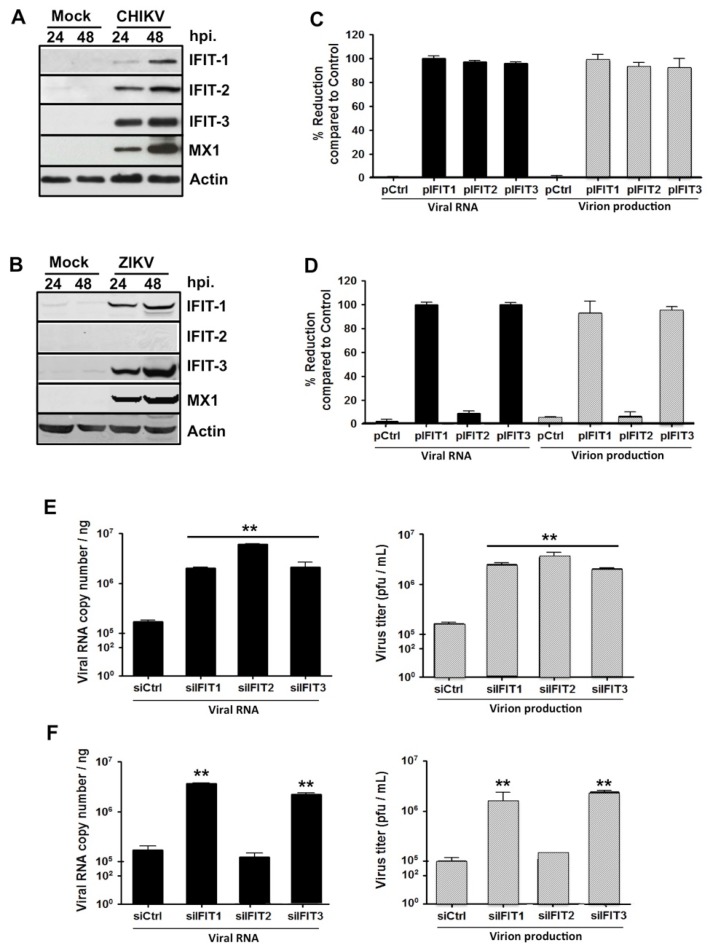
IFITs modulate CHIKV and ZIKV replication in HFF1 cells. (**A**) HFF1 cells were infected with CHIKV at a multiplicity of infection (MOI) of 1.0. At 24 and 48 hpi, infected cells were lysed with TETN-150 and analyzed by immunoblotting for the expression of IFIT1, IFIT2, IFIT3, MX1 and β-actin. (**B**) Similar to (**A**) but using ZIKV at a MOI of 1.0. For both (**A**,**B**), mock-infected cells were used as control. (**C**) CHIKV and (**D**) ZIKV RNA and infectious virus production were determined at 48 hpi in HFF1 cells transfected with an empty plasmid (pCtrl) or the same plasmid encoding IFIT proteins (pIFIT1, pIFIT2 and pIFIT3) by quantitative RT-PCR (black bars) and plaque assay (gray bars), respectively. The percentage of reduction as a function of the presence of each plasmid was calculated using the formula [1 − (R/C)] × 100, where C and R designate experimental values (RNA copy numbers or plaque numbers) in the presence of pCtrl and pIFIT, respectively. (**E**) CHIKV and (**F**) ZIKV RNA and infectious virus production were determined at 48 hpi in cells transfected with control siRNA (siCtrl) or siRNA, specific for IFIT (pIFIT1, pIFIT2 and pIFIT3) by real time RT-PCR (black bars) and plaque assay (gray bars), respectively. The data represent the mean values ± SD from three independent experiments. ** *p* < 0.01, as compared to siCtrl transfected cells.

Next, the IFIT proteins whose expression was induced in CHIKV- or ZIKV-infected HFF1 cells (Table 1) were selected for further investigation. Analysis of their expression levels by Western blotting analysis confirmed that the amounts of each of these proteins were increased in both CHIKV and ZIKV-infected human fibroblasts in a time-dependent manner (Table 1 and Figure 2A,B). As expected, the expression levels of IFIT2 in ZIKV infected cells were similar to those observed in mock-infected cells (Figure 2B), thus validating the results from the SILAC/MS analysis.

To study the functional role of IFITs proteins in the viral infection process, their genes were either overexpressed or their expression was suppressed in HFF1 cells using specific RNA silencing. The cells were subsequently infected with CHIKV or ZIKV, after which the synthesis of viral RNA and the production of viral particles were analyzed. Viral RNA levels and virion production of both viruses were almost totally abrogated in infected fibroblasts that overexpressed either IFIT1 or IFIT3 (Figure 2C,D), while viral RNA synthesis and virion production were significantly increased both in IFIT1- and IFIT3-silenced cells (Figure 2E,F). Consistent with our previous observations, the modulation of IFIT2 expression affected CHIKV but not ZIKV replication (Figure 2C,E). These results indicate that certain IFITs family members play a significant role in the regulation of the replication of these arboviruses.

### 2.3. SAMHD1 Expression Is Induced in Human Skin Fibroblasts Following CHIKV or ZIKV Infection

**Figure 3:**
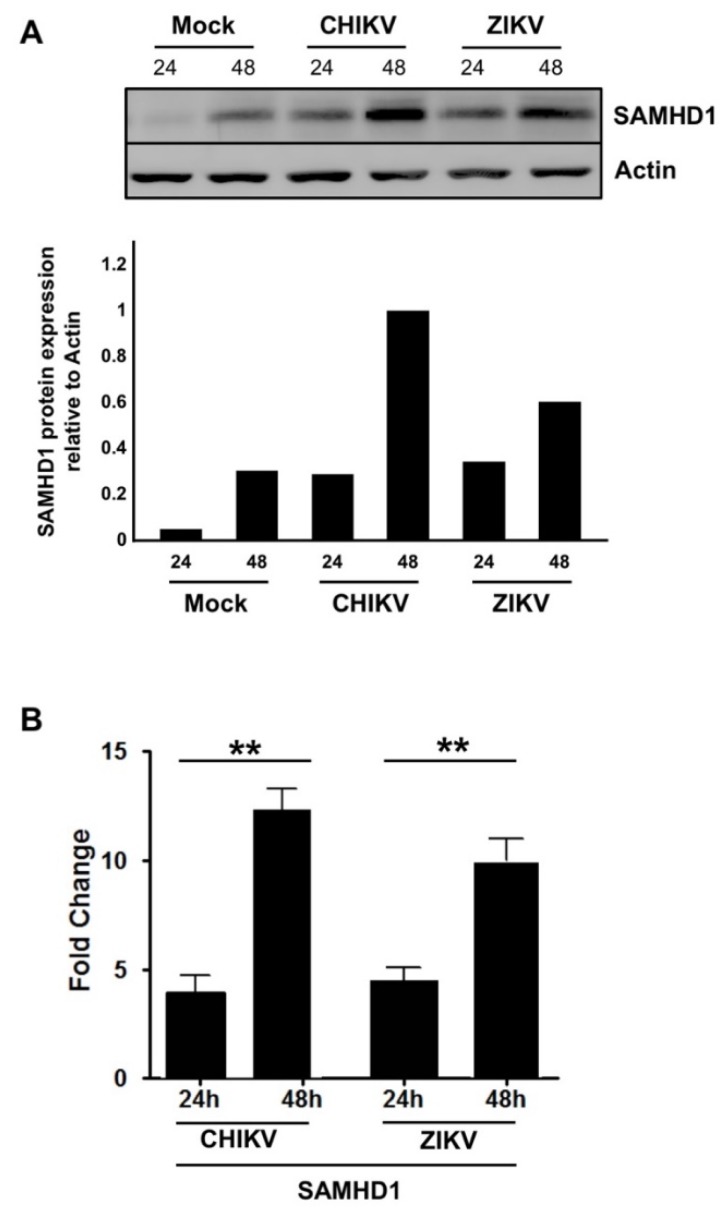
SAMHD1 expression is up-regulated upon CHIKV and ZIKV infection. HFF1 cells were infected with CHIKV or ZIKV at a MOI of 1.0. At 24 or 48 hpi, cells were lysed with TETN-150 and analyzed by immunoblotting using antibodies against SAMHD1 and β-actin Histograms represent relative values of SAMHD1/Actin expression shown in the immunoblot (**A**). Fold change in SAMHD1 expression in virus-infected cells in relation to Mock-infected cells (**B**). The data represent the mean values ± SD from three independent experiments. ** *p*-value < 0.01, as compared to mock-infected cells.

The results from the SILAC/MS analysis showed significantly increased expression of SAMHD1 in HFF1 cells following both CHIKV or ZIKV infection. To validate these results, immunoblotting analysis and RT-qPCR were performed in the infected cells. Western blotting analysis of CHIKV- and ZIKV-infected cells showed that in both cases SAMHD1 expression was up-regulated in a time-dependent manner (Figure 3A) and confirmed by RT-qPCR analysis (Figure 3B).

### 2.4. HIV-2 Vpx Protein-Induced SAMHD1 Degradation Inhibits CHIKV and ZIKV Replication

**Figure 4:**
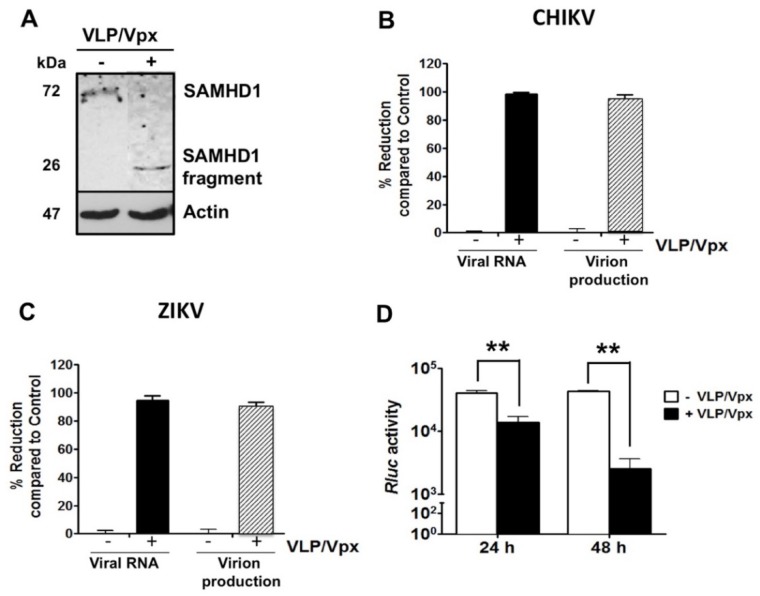
(**A**) Vpx-induced SAMHD1 degradation decreases CHIKV and ZIKV replication. Mock- (−) or VLP/Vpx-treated (+) HFF1 cells were infected with CHIKV for 48 h. Infected cells were lysed with TETN-150 and analyzed by immunoblotting using antibodies against SAMHD1 and β-actin. (**B**,**C**) Vpx-treated and mock-treated HFF1 cells were infected with CHIKV or ZIKV at MOI 1. After 48 h, intracellular viral RNA levels and infectious virus production were measured by real time RT-PCR (black bars) and plaque assay (gray bars), respectively. The percentage of reduction in the presence of Vpx was calculated using the formula [1 − (R/C)] × 100, where C and R designate experimental values (RNA copy numbers or plaque numbers) as a function of Mock- or VLP/Vpx-treated cells, respectively. (**D**) Stable Huh-7 cells harboring the CHIKV-NCT replicon were untreated or treated with VLP/Vpx. Twenty-four and 48 h post-treatment, cells were lysed and *Renilla Luciferase* (*Rluc*) activity was measured. Relative *Rluc* activity expressed by the CHIKV replicon represents the magnitude of CHIKV RNA replication. Values are normalized according to protein content of the cell extract and correspond to the mean of triplicates ± SD. The data represent the mean values ± SD from three independent experiments. ** *p*-value < 0.01, as compared to untreated cells.

SAMHD1 was initially identified as a restriction factor that inhibits HIV-1 replication in resting cells. It has been shown that SAMHD1 is degraded by the accessory protein Vpx, encoded by simian immunodeficiency virus (SIV) and HIV-2, but not by HIV-1 [22,23]. To study the effect of Vpx on endogenous SAMHD1 levels in human skin fibroblasts, the cells were incubated with virus-like particles (VLPs) that contain the Vpx protein of SIV [22], infected with CHIKV and the expression levels of SAMHD1 were determined by Western blotting analysis. At 24 hpi, SAMHD1 protein was not detectable in VLP/Vpx-treated cells (Figure 4A).

To assess whether Vpx-mediated SAMHD1 degradation affects replication of CHIKV and ZIKV, VLP/Vpx-treated or mock-treated HFF1 cells were infected with each virus. At 48 hpi, intracellular viral RNA levels, as well as titers of released virions, were significantly reduced in VLP/Vpx-treated cells, infected with CHIKV or ZIKV (Figure 4B,C), resulting in an almost complete lack of viral RNA synthesis and virion production. The effect of VLP/Vpx treatment on the post-entry steps of CHIKV infection cycle was confirmed using the Huh7-CHIKV replicon cell line that enables specific quantitation of viral RNA replication. The results of this analysis showed a highly significant decrease in *RLuc* activity which is directly proportional to CHIKV RNA replication, with a stronger inhibition at 48 h post-treatment (Figure 4D). Together, these data demonstrate that degradation of SAMHD1 by SIV Vpx inhibits CHIKV and ZIKV infection and that SAMHD1 impacts at least one of post-entry step of the CHIKV infection cycle.

### 2.5. SAMHD1 Enhances Both CHIKV and ZIKV Replication in HFF1 Cells

**Figure 5:**
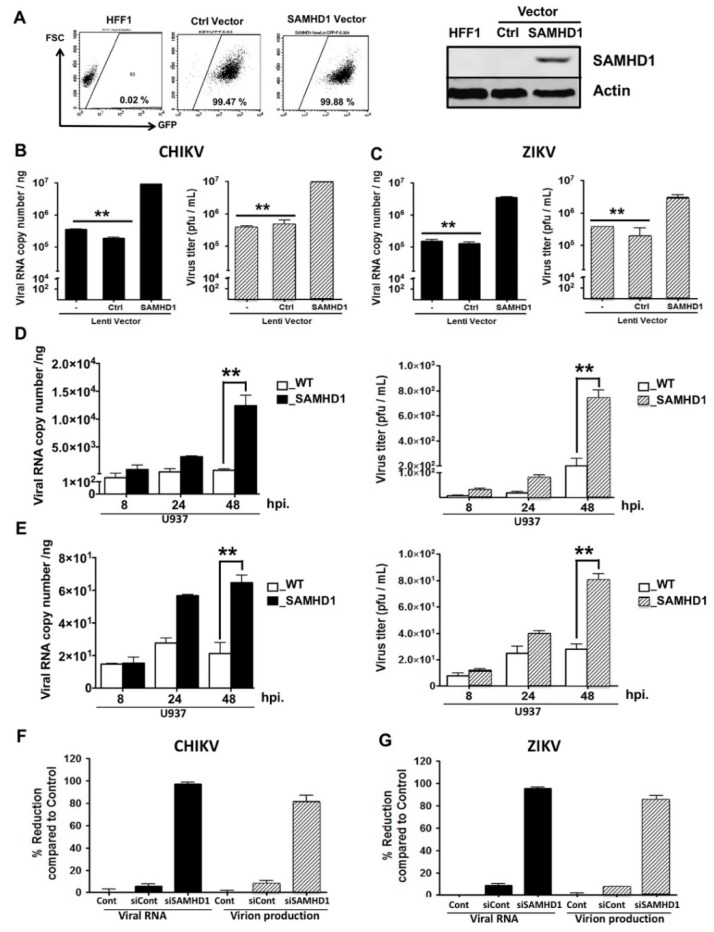
SAMHD1 over-expression enhances CHIKV and ZIKV replication. (**A**) HFF1 cells were transduced by either control (Ctrl)- or SAMHD1-GFP lentiviral vector. Transduced cells were sorted by a FACSAria III cytometer and obtained cell lines were validated for GFP expression by flow cytometry (10,000 single cell events in the analysis gate) and for SAMHD1 expression using Western blotting analysis. (**B**,**C**) Untreated (−), control transfected (Ctrl) or SAMHD1-(SAMHD1) transfected HFF1 cells were infected with CHIKV or ZIKV at MOI 1. At 48 hpi, intracellular viral RNA was quantified by RT-PCR (black bars); virus titers were measured using a plaque assay (gray bars). (**D**,**E**) Wild type U937 cells (WT) or U937 cells expressing SAMHD1, were infected with CHIKV or ZIKV at MOI 1. At 8, 24 and 48 hpi, the intracellular viral RNAs was quantified by RT-PCR (black bars); virus titers were measured by plaque assay (gray bars). (**F**,**G**) Amounts of CHIKV and ZIKV RNA and infectious virion production were determined at 24 hpi in HFF1 cells that were mock-transfected (Cont) or transfected, either with control siRNA (siCont) or siRNA specific for SAMHD1 (siSAMHD1). Expression of viral RNA and viral titers were quantified using real time RT-PCR (black bars) or plaque assay (gray bars), respectively. Percentage of reduction was calculated for each of the experimental conditions (siCont and siSAMHD1) using the formula [1 − (R/C)] × 100, where C and R designate experimental values (RNA copy numbers or plaque numbers) in untreated cells and siCont- or siSAMHD1-treated cells, respectively. The data represent the mean values ± SD from three independent experiments. ** *p*-value < 0.01 as compared to control cells.

The data obtained thus far indicate that SAMHD1 is an important pro-viral factor for both CHIKV and ZIKV. In contrast, however, SAMHD1 has been reported to inhibit the replicative process of HIV-1 and Hepatitis B virus by depleting the intracellular pool of dNTPs to levels below those required to complete the reverse transcription step [24,25,26,27,28]. Thus, SAMHD1 seems to exert opposite effects on viruses that use reverse transcription and arboviruses with a positive-strand RNA genome. To test this hypothesis, HFF1 cells that stably express either an irrelevant control or a SAMHD1 mRNA were generated following transduction with a GFP-containing lentiviral vector. Cell sorting by flow cytometry, based on the expression of GFP showed that almost all transduced cells expressed SAMHD1 transcripts. Expression of the SAMHD1 protein in these cells was also confirmed by Western blotting analysis (Figure 5A).

Next, parental and lentivirus-transduced HFF1 cells were either mock infected or infected with CHIKV or ZIKV. The presence of the SAMHD1-containing transgene resulted in a significant and prominent increase, close to 2 logs, both in intracellular viral RNA copy numbers and titers of CHIKV and ZIKV virions (Figure 5B,C). The impact of SAMHD1 over-expression on viral replication was further examined using wild-type U937 cells, that are not permissive for CHIKV [29] and its variant cell line that stably overexpresses SAMHD1. The results obtained with both virus strains were similar in that wild type U937 cells were poorly permissive for CHIKV and ZIKV. Increased viral RNA levels and viral titers were observed in the SAMHD1-expressing cells (Figure 5D,E). Finally, these results were corroborated by siRNA-mediated silencing of SAMHD1 in HFF1 cells. Intracellular viral RNA levels and titers of both viruses were strongly decreased upon SAMHD1 silencing. The observed inhibition in SAMHD1-silenced cells reached close to 99% and 95% for CHIKV and ZIKV, respectively. In contrast, the inhibition in control-siRNA treated cells was negligible (Figure 5F,G). Together, these results strongly suggest that SAMHD1 increases the replication of positive-strand RNA viruses such as CHIKV and ZIKV.

## 3. Discussion

Arboviruses, e.g., CHIKV and ZIKV, belonging to the alphavirus and flavivirus family, respectively, are transmitted by the genus *Aedes* to humans via a bite in the skin during blood feeding. The ensuing viral entry, RNA replication and release of viral particles is the result of complex interactions between these viruses and their human host cells. The identification of host proteins that are differentially expressed during an infection with CHIKV and ZIKV will undoubtedly provide a better understanding of the processes leading to the pathology caused by these viruses and may pave the way to the development of efficient therapeutic intervention. To that aim, we have determined the proteomic profiles of CHIKV and ZIKV-infected human skin fibroblasts that form the main site of entry for arboviruses following the bite of an infected mosquito [20,21]. The proteomics analysis of both arbovirus-infected human cutaneous fibroblast cell line HFF1 resulted in the identification of 2132 and 2696 proteins whose expression was modulated in CHIKV- and ZIKV-infected cells, respectively. Sixteen proteins, nine of which were common for cells infected with both viruses, were significantly over-expressed, as compared to the non-infected control cells and most were encoded by ISGs.

Both alphaviruses and flaviviruses trigger the IFN type I signaling pathway, leading to the transcription of ISGs, whose protein products prevent or suppress the infection of these pathogens [30]. CHIKV was found to induce the expression of IFIT1, -2 and -3 proteins. IFITs are ISGs that can inhibit viral replication through multiple mechanisms, including suppression of translation initiation [31], binding of uncapped or incompletely capped viral RNA [32,33], as well as sequestration of viral proteins or RNAs in the cytoplasm of the host cell [34,35]. It has furthermore been shown that IFITs have specific antiviral functions in response to human parainfluenza virus type 3 infection [36] and, similarly, that IFIT1 interferes with the translation and replication of several alphaviruses [37]. Our results confirm and extend these observations by showing that the forced expression of IFIT1, IFIT2 or IFIT3 in human fibroblasts completely inhibited the replication of CHIKV and that the individual knock-down of each of these genes facilitated viral infection.

SAMHD1 is a deoxynucleoside triphosphate triphosphohydrolase protein with a sterile α-motif (SAM) and an HD domain that harbors the active site of the protein [38]. It has been identified as a HIV-1 restriction factor and has been shown to be degraded by the HIV accessory factor Vpx through a proteasome-dependent mechanism [24,39,40]. Several recent studies have investigated the role of the SAMHD1 protein in inhibiting virus infectivity. It has been proposed that the inhibitory effect of SAMHD1 on HIV-1 replication is due to its dNTPase activity [41] or, alternatively, that SAMHD1, or an associated protein, could restrict HIV-1 replication through its RNase activity [42,43]. These findings notwithstanding, no information is as yet available regarding the role of SAMHD1 in arbovirus infections. In contrast to the results from the above-mentioned studies that have underscored the role of SAMHD1 as a restriction factor able to inhibit the replication of reverse transcription-required viruses [27,28,44,45,46,47], the present study is the first to demonstrate that SAMHD1 promotes CHIKV and ZIKV replication. First, Vpx/VLP-mediated SAMHD1 degradation was found to cause a prominent decrease in the replication of and virion production by both viruses. Secondly, the same treatment reduces replication of CHIKV replicon. Finally, the over-expression of SAMHD1 facilitates CHIKV and ZIKV replication in several cell lines. The contrast between our findings and the previously reported evidence with respect to the anti-viral functions of SAMHD1 is most likely due to differences in the replication process used by these viruses: CHIKV and ZIKV, unlike HIV-1 and HBV, do not require the reverse transcription step for their replication. In this respect, a successful HIV replication cycle requires the presence of dNTP, which is not the case for arboviruses. Support for the proviral activity of SAMHD1 comes from its capacity to suppress innate immune responses. Indeed, it was shown that SAMHD1 interacts with the inhibitor-κB kinase ε (IKKε) and IFN regulatory factor 7 (IRF7), leading to the suppression of the IFN-I induction pathway by reducing IKKε-mediated IRF7 phosphorylation. Moreover, SAMHD1 also inhibits NF-κB activation by interacting with NF-κB1/2, thereby reducing phosphorylation of the NF-κB inhibitory protein IκB [48].

In conclusion, our work highlights the crucial role of IFITs family members in the regulation of CHIKV and ZIKV replication. Our data also provide novel evidence on the role of SAMHD1 in arbovirus infection of human skin cells. The modulation of SAMHD1 expression found in our study might be a strategy of CHIKV and ZIKV to facilitate their replication. The results reported here therefore open new perspectives for the study of the precise role of SAMHD1 in arbovirus infection.

## 4. Materials and Methods

### 4.1. Cells and Viruses

C6/36 *Ae*. *albopictus* cells (ATCC^®^ CRL-1660™), used for propagation of the CHIKV and ZIKV strains, were grown at 28 °C in Dulbecco’s modified Eagle’s medium (DMEM; Invitrogen, Cergy Pontoise, France), supplemented with 10% fetal calf serum (FCS; Lonza, Basel, Switzerland) at 28 °C, as previously described [21]. The human skin fibroblast cell line HFF1 (ATCC^®^ SCRC-1041™) and U937 (ATCC^®^ CRL-1593.2™) cells were cultured in DMEM, supplemented with 15% and 10%, respectively.

The low-passage-number of the LR2006_OPY1 strain (isolated from a viremic patient in La Réunion Island in 2006) was a kind gift from Dr. Philippe Desprès (PIMIT, Inserm U1187, St Clotilde). The clinical isolate PF-25013-18 of ZIKV has been previously described [21]. Both virus strains were grown in C6/36 cells. Lentiviral virus like particles (VLPs) containing Vpx (VLPs/Vpx) were generated as previously described [49].

### 4.2. Antibodies, Plasmids and Reagents

Monoclonal antibody anti-SAMHD1 antibody was purchased from Abcam (Paris, France). Plasmids encoding IFIT proteins were purchased from Addgene (Watertown, NY, USA). Monoclonal antibody anti-IFIT1, IFIT2, IFIT3, MX1 and β-actin antibodies were purchased from GeneTex (Souffelweyersheim, France).

### 4.3. SILAC Labeling and Virus Infection

HFF1 cells were cultured in SILAC DMEM (PAA, Österreich, Austria), 10% dialyzed FCS (Gibco, Loughborough, UK), 0.280 mM arginine, 0.398 mM lysine, 0.5 mM proline, 10 mM HEPES, 2 mM l-glutamine, 100 IU penicillin and 100 μg/mL streptomycin for >5 cell doublings to ensure complete incorporation of the labeled amino acids. Arginine to proline conversion was not observed under these labeling conditions. The SILAC light medium (L) was supplemented with Arg ^12^C_6_^14^N_4_ and Lys ^12^C_6_^14^N_2_ (Sigma, Zwijndrecht, The Netherlands), the SILAC heavy medium (H) was supplemented with Arg ^13^C_6_^15^N_4_ and Lys ^13^C_6_^15^N_2_ (Cambridge Isotope Laboratories, Cambridge, MA, USA).

HFF1 cells were seeded in six-well plates and grown to a 70–80% confluence. The cultures were rinsed twice with PBS and the cells were incubated with either CHIKV or ZIKV at MOI of 8 for 2 or 1.5 h, respectively, at 37 °C while gently agitating the plates. Then, the inoculum was removed, the cells were washed three times with PBS and SILAC DMEM supplemented with 15% FCS was added to each well after which the plates were incubated at 37 °C and 5% CO_2_. Infected and mock-infected cells were lysed at 48 hpi in 4% SDS, 0.1 M Tris pH 7.6.

### 4.4. Mass Spectrometry Analysis

Proteins were separated on SDS-PAGE gels (10% polyacrylamide; Mini-Protean TGX Precast Gels; Bio-Rad, Marnes-la-Coquette, France) and stained with Page Blue Stain (Fermentas, Burlington, Canada). Gel lanes were cut into 9 gel pieces and destained by three washes in 50% acetonitrile and 50 mM triethylammonium bicarbonate (TEABC). After protein reduction (with 10 mM dithiothreitol in 50 mM TEABC at 56 °C for 45 min) and alkylation (55 mM iodoacetamide TEABC at RT for 30 min), proteins were in-gel digested using trypsin (500 ng/band; Gold; Promega, Fitchburg, USA).

Peptides were analyzed online by nano-flow HPLC-nanoelectrospray ionization using a LTQ-orbitrap Elite mass spectrometer (Thermo Fisher Scientific, MA, USA) coupled to a nano-LC system (U3000 Dionex, Thermo Fisher Scientific). Desalting and preconcentration of samples were performed on-line on a Pepmap^®^ precolumn (0.3 × 10 mm; Dionex). A gradient consisting of 0–40% B in A for 80 min (A: 0.1% formic acid, 2% acetonitrile in water, and B: 0.1% formic acid in acetonitrile) at 300 nL/min, was used to elute peptides from the capillary reverse-phase column (0.075 × 150 mm, Pepmap^®^, Dionex). Data were acquired using the Xcalibur software (version 2.2). A cycle of one full-scan mass spectrum (400–2000 *m*/*z*) at a resolution of 120,000 (at 400 *m*/*z*), followed by 20 data-dependent MS/MS spectra (LTQ) was repeated continuously throughout the nanoLC separation. Raw data analysis was performed using the MaxQuant software (version 1.5.0.0) with standard settings [50]. Used database consist of Human, ZIKV and CHIKV entries from Uniprot and 250 contaminants (MaxQuant contaminant database). Relative protein quantifications were calculated on the median SILAC ratios. For further analysis, only proteins with at least two peptides were considered. Determination of significant proteins was assessed using significance A, using Perseus (version 1.5.0.0) on the log2 ratio after elimination of contaminant and reverse entries.

### 4.5. Bioinformatics Analysis

We selected as significantly up-regulated proteins all those proteins having inversed ratios in H/L and L/H conditions and a detection *p*-value smaller than 0.05 in both conditions.

We used the g:Profiler tool [51] (November 2015, version: r1477_e82_eg29) to assess the over-representation of Gene Ontology and biological pathways annotations (KEGG and Reactome) among the up-regulated proteins. We considered as significant those annotations having an adjusted *p*-value < 0.025 after Benjamini–Hochberg multiple testing correction. We built a human functional interaction network among CHIKV and ZIKV up-regulated proteins by using the STRING database v10 [52]. We selected all the functional interactions having a medium confidence score (i.e., equal or greater than 0.4).

### 4.6. VLPs/Vpx Treatment

HFF1 cells were pre-incubated with vehicle or VLPs/Vpx at the MOI 2.0 for 24 h. Then, treated cells were infected with CHIKV or ZIKV for 2 or 1.5 h before further experiments. The cells were maintained with VLPs/Vpx throughout the infection.

### 4.7. Infection

Cells were grown to 70% confluence in six-well plates. On the day of infection, cells were washed twice with phosphate-buffered saline before inoculated with viruses at the desired MOI for different periods. Negative control cells were prepared by incubating the cells with culture supernatant from uninfected C6/36 cells.

### 4.8. Small Interfering RNA (siRNA) Transfection

Cells were transiently transfected with an 80 nM final concentration of small interfering RNAs (siRNAs) targeting SAMHD1 by using Lipofectamine^®^ 2000 (Thermo Fisher Scientific). For IFITs proteins, a 25 nM final concentration of siRNAs was used. After 24 h, cells were mock-infected or infected with CHIKV or ZIKV. The following pools of siRNAs (ON-TARGETplus SMARTpool) used in this study were from Dharmacon: siRNA pools for SAMHD1 (L-013950-01), IFIT1 (L-019616-00), IFIT2 (L-012582-02) and IFIT3 (L-017691-00). A nontargeting pool (NT) was used as a negative control.

### 4.9. Generation of SAMHD1-Expressing Cell Line

HFF1 cells were plated at a density of 5 × 10^4^ cells per well in 24 wells plate. After incubate at 37 °C with 5% CO_2_ for 24 h, Control-GFP-lentivirus and SAMHD1-GFP-lentivirus were transduced according to the manufacturer’s protocol (ABM^®^, Richmond, BC, Canada). Transduced cells were sorted using a FACSAria IIIr^®^ cytometer (Becton and Dickinson Biosciences, San Jose, CA, USA) and expanded in DMEM supplemented with 15% FCS and 2.5 μM puromycin. GFP expression was determined by flow cytometry using a FACSCalibur^®^ flow cytometer and Cell Quest software. U937 cells stably expressing SAMHD1 were generated as previously described [53].

### 4.10. Western Blotting Analysis

Cells were lysed on ice in TETN-150 supplemented a cocktail of protease inhibitors (Sigma) [50]. Protein concentration was determined by bicinchoninic acid (BCA) assay (Thermo Scientific, Saint Herblain, France). Equal amounts of proteins were mixed with Laemmli sample loading buffer, subjected to SDS-PAGE and electrotransferred onto a nitrocellulose membrane. The membrane was blocked with 0.05% Tween 20 in PBS (PBST) containing 5% skim milk for 1 h at RT, incubated overnight at 4 °C with desired primary antibody, washed three times with PBST, and subsequently incubated for 1 h at RT with horseradish peroxidase-coupled secondary antibodies (Cell Signaling, MA, USA) in PBST. The membrane was washed three times, and proteins were detected by chemiluminescence using a SuperSignal West Pico chemiluminescent substrate kit (Thermo Scientific). The membrane was then stripped and re-probed with an anti-β-actin antibody to ensure that equivalent levels of protein were loaded in each lane.

### 4.11. Viral RNA Quantification and Gene Expression by Real Time RT-PCR

Total RNA was extracted using Tri reagent (Sigma, Saint Quentin Fallavier, France) according to the manufacturer’s protocol. The RNA pellet was resuspended in 25 μL of RNase-free distilled water and stored at −80 °C. The RNA was used for reverse transcription using Moloney murine leukemia virus (M-MLV) reverse transcriptase (Promega, Charbonnieres, France) according to the manufacturer’s instructions. The reaction was carried out using 1 μg total RNA as template for the normalization of viral RNA to the amount of total RNA. The MaximaTM Probe/ROX qPCR Master Mix (2×) (Thermo Scientific) was used in qPCR experiment. Each reaction of 25 μL contained 400 nM of each primer, 200 nM of specific probe and 1× Maxima^TM^ Probe/ROX qPCR Master Mix. Primers and probe sequences are listed in Table S4. The amplification conditions were 95 °C for 10 min followed by 45 amplification cycles of 95 °C for 15 s, 60 °C for 20 s and 72 °C for 30 s. The reactions were performed in an Applied Biosystem 7300 system. Real time data were analyzed using the SDS software (Thermo Fischer Scientific). Viral RNA was quantified by comparing the sample’s threshold cycle (*C*t) values with each virus RNA standard curve which was obtained as previously described [20,21].

### 4.12. CHIKV Replicon Cell Line Based Assay

Huh-7 cells stably transfected with the CHIKV-NCT (NonCytotoxic) replicon, were used to test the effects of SAMHD1 to CHIKV replication. CHIKV replicon cells were plated in six-well plates, treated with VLPs/Vpx and incubated at 37 °C and 5% CO_2_. After 24 and 48 h of incubation, Renilla Luciferase (*Rluc*) activity, expressed by the CHIKV replicon, was detected using the Renilla Luciferase assay (Promega, Charbonnière, France) according to the manufacturer’s instructions. The luminescence signal, proportional to the CHIKV’s RNA replication, was then measured using the Modulus microplate luminometer (Turner BioSystems, Sunnyvale, CA, USA).

### 4.13. Plaque Assay

Vero cells, grown to 70–80% confluence, were incubated with four separate, ten-fold, dilutions of viral supernatant and cultured at 37 °C for 2 h. Then, a mix of nutriment solution with agar (Lonza) was added and the cells were maintained at 37 °C for 6 days. For plaque counting, the cells were fixed with 3.7% formaldehyde and stained with 0.5% Crystal violet in 20% ethanol.

### 4.14. Data Analysis and Statistical Methods

All data are presented as means ± standard deviation (SD). The Student’s *t* test was used to determine the statistical significance. For all experiments, statistical significance was accepted at *p* < 0.05.

## Figures and Tables

**Table 1:** Proteins with significantly up-regulated expression in CHIKV- and ZIKV-infected cells.

Proteoform Name	Gene Name	Uniprot ID	Average log2 Ratio
**Chikungunya virus infected cells proteins**			
Interferon-induced GTP-binding protein Mx1	MX1 *	P20591	5.043
Interferon-induced protein with tetratricopeptide repeats 3	IFIT3 *	O14879	3.591
Interferon-induced protein with tetratricopeptide repeats 1	IFIT1 *	P09914	3.449
Interferon-induced protein with tetratricopeptide repeats 2	IFIT2	P09913	2.954
Ubiquitin-like protein ISG15	ISG15 *	P05161	2.580
Probable ATP-dependent RNA helicase DDX58	DDX58 *	O95786	2.444
Signal transducer and activator of transcription 1-alpha/beta	STAT1 *	P42224	1.659
2′-5′-oligoadenylate synthase 3	OAS3 *	Q9Y6K5	1.626
Deoxynucleoside triphosphate triphosphohydrolase SAMHD1	SAMHD1 *	Q9Y3Z3	1.208
HLA class I histocompatibility antigen, B-45 alpha chain	HLA-B	P30483	1.119
Cytosol aminopeptidase	LAP3	P28838	1.049
Interferon-induced 35 kDa protein	IFI35	P80217	1.015
E3 ubiquitin-protein ligase RNF213	RNF213	Q63HN8	0.895
Gamma-interferon-inducible protein 16	IFI16	Q16666	0.878
Double-stranded RNA-activated protein kinase	EIF2AK2 *	P19525	0.838
HLA class I histocompatibility antigen, A-69 alpha chain	HLA-A	P10316	0.752
**Zika virus infected cells proteins**			
Interferon-induced GTP-binding protein Mx1	MX1 *	P20591	5.622
Interferon-induced protein with tetratricopeptide repeats 1	IFIT1 *	P09914	3.362
Interferon-induced protein with tetratricopeptide repeats 3	IFIT3 *	O14879	3.076
Ubiquitin-like protein ISG15	ISG15 *	P05161	2.430
2′-5′-oligoadenylate synthase 3	OAS3 *	Q9Y6K5	2.190
Poly [ADP-ribose] polymerase 9	PARP9	Q8IXQ6-2	2.118
Phospholipid scramblase 1	PLSCR1	O15162	2.085
Probable ATP-dependent RNA helicase DDX58	DDX58 *	O95786	1.974
Deoxynucleoside triphosphate triphosphohydrolase SAMHD1	SAMHD1 *	Q9Y3Z3	1.739
Signal transducer and activator of transcription 1-alpha/beta	STAT1 *	P42224	1.440
Synaptopodin-2	SYNPO2	Q9UMS6	1.415
E3 ubiquitin-protein ligase DTX3L	DTX3L	Q8TDB6	1.397
2′-5′-oligoadenylate synthase 1	OAS1	P00973	1.135
E3 ubiquitin-protein ligase TRIM21	TRIM21	P19474	1.085
Double-stranded RNA-activated protein kinase	EIF2AK2 *	P19525	1.071
Interferon-induced GTP-binding protein Mx2	MX2	P20592	0.993

* Common proteins for CHIKV and ZIKV.

**Table 2:** Functional annotations: Significant pathways identified from CHIKV- and ZIKV-infected cells.

**Chikungunya Virus**		
**Biological Processes**	**GO Term**	**Corrected *p*-Value**
**GO ID**		
GO:0060337	Type I interferon signaling pathway	9.94 × 10^−21^
GO:0051607	Defense response to virus *	3.53 × 10^−16^
GO:0048525	Negative regulation of virus infection process *	1.37 × 10^−10^
GO:0045071	Negative regulation of viral genome replication	4.25 × 10^−10^
GO:0031348	Negative regulation of defense response	0.0000326
GO:0035455	Response to interferon-alpha *	0.000153
GO:0045088	Regulation of innate immune response *	0.000212
GO:0060333	Interferon-gamma-mediated signaling pathway	0.000249
GO:0002698	Negative regulation of immune effector process	0.000407
GO:0043330	Response to exogenous dsRNA *	0.00281
GO:0050688	Regulation of defense response to virus	0.00602
**KEGG**	**Pathway Name**	**Corrected *p*-Value**
**Pathway ID**		
KEGG:05168	Herpes simplex infection	4.12 × 10^−8^
KEGG:05160	Hepatitis C *	0.0000275
KEGG:05162	Measles *	0.0000286
KEGG:05164	Influenza A	0.000101
KEGG:05169	Epstein-Barr virus infection	0.00553
**Reactome**	**Pathway Name**	**Corrected *p*-Value**
**Pathway ID**		
REAC:913531	Interferon Signaling *	5.41 × 10^−19^
**Zika Virus**		
**Biological Processes**	**GO Term**	**Corrected *p*-Value**
**GO ID**		
GO:0051607	Defense response to virus *	5.74 × 10^−16^
GO:0071357	Cellular response to type I interferon	2.77 × 10^−15^
GO:0048525	Negative regulation of viral process *	3.85 × 10^−15^
GO:1903901	Negative regulation of viral life cycle	7.82 × 10^−13^
GO:0035455	Response to interferon-alpha *	0.000000633
GO:0034341	Response to interferon-gamma	0.0000822
GO:0043330	Response to exogenous dsRNA *	0.00456
GO:0045088	Regulation of innate immune response*	0.00899
**KEGG**	**Pathway Name**	**Corrected *p*-Value**
**Pathway ID**		
KEGG:05160	Hepatitis C *	0.0104
KEGG:05162	Measles *	0.0108
**Reactome**	**Pathway Name**	**Corrected *p*-Value**
**Pathway ID**		
REAC:913531	Interferon Signaling *	1.22 × 10^−15^

* Common pathways identified from CHIKV- and ZIKV-infected cells.

## Supplementary Materials

Supplementary Materials can be found at https://www.mdpi.com/1422-0067/20/7/1695/s1. Table S1: SILAC analysis raw data of CHIKV-infected cells, Table S2: SILAC analysis raw data of ZIKV-infected cells, Table S3: Functional annotation of CHIKV- and ZIKV-infected cells, Table S4: Primers and probes for viral detection used in this study.
