# Supplementary material for: SAMHD1 Enhances Chikungunya and Zika Virus Replication in Human Skin Fibroblasts
Source: Int J Mol Sci. 2019 Apr 5;20(7):1695. doi: 10.3390/ijms20071695 (PMC6480247; doi:10.3390/ijms20071695)
Supplement: Supplementary file 1 [file ijms-20-01695-s001.zip › Supplemetary-IJMS/Table_S4_Primers and probes for viral detection used in this study.docx]

**Supplementary Table S4**

Primers and probes for viral detection used in this study.

| Virus | Primer | Sequence (5’->3’) |
| --- | --- | --- |
| CHIKV | CHIKV-F | 5’AAGCT(CT)CGCGTCCTTTACCAAG3’ |
|  | CHIKV-R | 5’CCAAATTGTCC(CT)GGTCTTCCT3’ |
|  | CHIKV-P | 5’CCAATGTC(TC)TC(AC)GCCTGGACACCT3’ |
| ZIKV | ZIKV-F | 5’TTGGTCATGATACTGCTGATTGC3’ |
|  | ZIKV -R | 5’CCTTCCACAAAGTCCCTATTGC3’ |
|  | ZIKV -P | 5’CGGCATACAGCATCAGGTGCATAGGAG3’ |

| Gene | Primer | Sequence (5’->3’) |
| --- | --- | --- |
| SAMHD1 | SAMHD1-F | 5’AAAACCAGGTTTCACAACTTCTGC3’ |
|  | SAMHD1-R | 5’TGCGGCATACAAACTCTTTCTGT3’ |
